# Dunn

**Published:** 1869-04-01

**Authors:** 


### *Death of Dr. A. A. Dunn.*

At a regular meeting of the Chicago Medical Society, held on Friday evening, the 12th inst., the following report was adopted:

The undersigned committee to whom was referred the duty of expressing the sentiments of this society regarding the late A. A. Dunn, M.D., of this city, would respectfully report:

*Resolved*, That Dr. Dunn, by his natural intellectual endownents, his education, his professional skill and integrity, his ardent support of medical societies and ethics, had won the esteem and confidence of all the members of the profession who knew him; while the addition of a uniform Christian deportment, and a self-sacrificing patriotism, had added equally the respect and warm attachment of all classes of community.

*Resolved*, That by his death, the profession and society have lost one of their most respected and useful members, the nation one of its true heroes and defenders, and this society one we all esteemed as a brother.

*Resolved*, That a copy of the foregoing expressions of sentiment be communicated to the family of the deceased, and to the medical journals and daily papers of this city.

N. S. Davis,

T. D. Fitch,

A. Groesbeck, } *Committee.*

